# Spatial dynamics of the 1918 influenza pandemic in England, Wales and the United States

**DOI:** 10.1098/rsif.2010.0216

**Published:** 2010-06-23

**Authors:** Rosalind M. Eggo, Simon Cauchemez, Neil M. Ferguson

**Affiliations:** MRC Centre for Outbreak Analysis and Modelling, Imperial College London, Department of Infectious Disease Epidemiology, St Mary's Campus, London W2 1PG, UK

**Keywords:** gravity model, spatial interaction, influenza pandemic, density dependence

## Abstract

There is still limited understanding of key determinants of spatial spread of influenza. The 1918 pandemic provides an opportunity to elucidate spatial determinants of spread on a large scale.

To better characterize the spread of the 1918 major wave, we fitted a range of city-to-city transmission models to mortality data collected for 246 population centres in England and Wales and 47 cities in the US. Using a gravity model for city-to-city contacts, we explored the effect of population size and distance on the spread of disease and tested assumptions regarding density dependence in connectivity between cities. We employed Bayesian Markov Chain Monte Carlo methods to estimate parameters of the model for population, infectivity, distance and density dependence. We inferred the most likely transmission trees for both countries.

For England and Wales, a model that estimated the degree of density dependence in connectivity between cities was preferable by deviance information criterion comparison. Early in the major wave, long distance infective interactions predominated, with local infection events more likely as the epidemic became widespread. For the US, with fewer more widely dispersed cities, statistical power was lacking to estimate population size dependence or the degree of density dependence, with the preferred model depending on distance only. We find that parameters estimated from the England and Wales dataset can be applied to the US data with no likelihood penalty.

## 1. Introduction

Spatially explicit models are critical to understanding the spread of infectious diseases through populations and to better inform policy aimed at controlling that spread. Indeed, recent outbreaks of communicable diseases in human populations have triggered a series of studies addressing the spread of directly transmissible infections at a country level [1–5]. Identifying a possible backbone of high probability transmission paths through populations may underpin the development of effective interventions to curtail spread on the population network [6]. For example, in human diseases, spatial models and microsimulations can quantify the possible role of border control, quarantine or transport reductions in curtailing local and international spread [2–4,6–11]. Spatial microsimulation models like these are critical to making effective policy decisions. Spatial models also allow limited control resources to be used where they might be most effective. For instance, during the initial stages of an outbreak of a new virus, disease incidence tends to occur in spatial clusters with occasional long-range infection events [10]. As case numbers increase in the start location, the frequency of long-range infection events increases. This spatial pattern was seen during the early stages of the 2009 influenza pandemic in Mexico, leading to local foci seeded by long-range interactions to other countries [12,13].

However, those epidemic models rely on a set of structural assumptions that need to be validated from data. A basic assumption of many spatially explicit transmission models is that flows between urban centres are a function of the distance between them and their attributes, most notably residential or worker population sizes [14,15], resulting in a so-called gravity model. However, for human diseases, little work has been done to validate the underlying assumption that human travel patterns are predictive of the spatial spread of diseases.

Early models of spatial coupling in ecology assumed that connectivity between populations was inversely related to the distance between them [16]. For people (and most animals) distance-based coupling is too simplistic an assumption. Movement between large population centres is disproportionately more frequent than between smaller ones [17]. Xia *et al.* [18] found that a distance-only model for spread of measles in the UK was a poor fit to weekly measles data from England and Wales from 1944 to 1967. The gravity model used in that study measures connectivity between population centres as a function of distance and a function of the population sizes of the origin and destination cities. However, measles is a childhood disease, and so the spatial dependencies of the host are different than for infections that affect both adults and children, such as pandemic influenza. Gravity models and other spatial interaction models allow understanding of the movement of populations from one location to another in the absence of movement data. Models, once validated, can predict modifications in connectivity when populations grow or shrink, when workflows vary owing to economic changes or if restrictions are imposed on one city and not others. This is in contrast to movement surveys, which are context-specific and provide a snapshot of the movement habits of a population.

The strength of connection between cities may be density independent, that is, the sum of connectivity of a city to all its neighbours does not depend on the number of neighbours that city has. In contrast, density-dependent connectivity links two cities at a strength solely determined by the sizes of those cities and their distance apart, so that the total connectivity of any one city scales with the number of close neighbours. Density-independent transmission gives a total force of infection, which is independent of the remoteness of the population, whereas density-dependent transmission will cause populations with many neighbours to experience a higher force of infection than those cities that have few neighbours. Thus, a density-dependent model will predict that isolated populations are less likely to become infected than populations with many neighbours, or few very large neighbours. The concepts of density dependence/independence have not only been used to model interactions between cities; they have also been used extensively in individual based models of disease spread. Most past studies tend to assume either density dependence (e.g. for animal epidemics; [19,20]), or density independence (e.g. for most human diseases) [3,4]. Here, we explore the extent to which city-to-city (rather than individual-to-individual) contacts are density independent by constructing a model that can capture intermediate levels of density dependence.

In this paper, we analyse mortality datasets from England and Wales and the United States from 1918 to 1919 to examine the pattern of spatio-temporal spread and the extent to which gravity models can reproduce observed trends. The 1918 pandemic constitutes a rare example of a well-documented epidemic in a largely susceptible human population, where the high mortality gives a clear incidence signal, and is therefore a rare opportunity to validate models of epidemiologically relevant geographic coupling. We examine the effect of city-specific characteristics (e.g. location, distance from other cities, population size and the number of influenza-related deaths) on the pattern of spread seen. We also investigate the impact of the distribution of cities in each country. The analysis provides further insight on the spatial variation in the spread of the 1918 influenza pandemic at a country level, much of which remained unexplained in past studies [18,21–23].

## 2. Material and methods

### 2.1. Data

The 1918 pandemic H1N1 virus appears to have entered the general population of the UK and US in the spring of 1918 causing a reportedly mild disease [24]. This early wave was associated with increased mortality, but was probably only noticed because influenza is rare in summer. This epidemic waned later in the summer, but infection reappeared in the autumn with much increased mortality. It is unclear whether the viruses causing the spring and summer waves were closely related, but there is increasing evidence that the spring wave gave immunological protection against the autumn wave at a population level [25,26]. By September 1918, the pandemic was a prominent global phenomenon. The autumn wave was virtually universal, albeit with some variation between countries in precise timing. In both the UK and the US, a third wave of influenza occurred in early 1919 although with greater heterogeneity in mortality rates between cities [24,27,28]. US cities had more variation in the severity of the major wave than the UK, probably in part because some enacted more stringent non-pharmaceutical interventions to mitigate the epidemic [27,28]. The third wave was less pronounced in US cities than in England and Wales, again perhaps partly because of the effect of interventions.

### 2.2. England and Wales

**Figure 1.**
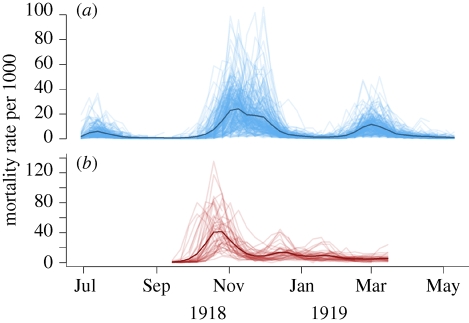
The two mortality incidence datasets. The darker line is a non-population weighted mean of all cities. (*a*) 246 population centres in England and Wales; (*b*) 47 cities in the US.

The England and Wales dataset shown in figure 1*a* was published in the Supplement to the 81st Registrar General Report [29]. It provides weekly death counts and annualized mortality rates per 1000 from 83 county boroughs, 84 municipal boroughs, 71 urban districts and three unclassified urban centres in England and Wales, for a 46 week interval, 29 June 1918–10 May 1919. The first 10 weeks are designated as wave 1, the next 19 as wave 2 and the last 17 as wave 3. Our analysis focuses on the second wave because it occurred in all cities (unlike wave 3) and because recording of mortality had begun in all cities before its arrival (unlike wave 1). In addition, reporting of influenza and influenza-related mortality changed between the first and second waves. Point locations of all urban centres were determined from the current or historical records, with Euclidean distance used to quantify inter-centre separation. Further information is given in the electronic supplementary material.

### 2.3. United States of America

We compiled a US city dataset (figure 1*b*) from five publications reporting the Weekly Health Index as collated by the Bureau of the Census [24,30–33]. It covers the period 14 September 1918 to 15 March 1919 and contains weekly pneumonia and influenza death counts for 47 cities in the US. The US data therefore covers a period of two waves, with not all cities experiencing the later wave. There is very good agreement between different sources where they overlap. We used the Euclidean distance between cities (accounting for curvature of the Earth) to measure separation. Further information is given in the electronic supplementary material.

### 2.4. Reconstructing the week of infection

The analysis requires an estimate of when each city became infected to allow potential sources of that infection to be identified. For each city infected in week *t*, the candidate infectors are those infected in any week before *t*. We define the infection week of city *i*, *t*_*i*_, to be the first that meets a set of conditions on mortality in weeks *t_i_* + 1, *t_i_* + 2 and *t_i_* + 3. We use mortality values ahead of *t*_*i*_ to include the time from infection to death. A week could be designated the infection week if either (or both) of two sets of criteria were met for mortality in the following weeks. The first set of criteria required the mortality rate in week *t_i_* + 1 to be above a certain threshold, to have increased in *t_i_* + 2 and to be above a higher threshold in *t_i_* + 3. These criteria are intended to ensure that the epidemic in that city is patently increasing. The second set of criteria was designed to capture cities where there was a rapid onset of increased influenza-related mortality. They therefore used a higher threshold on mortality in week *t_i_* + 1, but less strict conditions on rate of growth in the following two weeks. The week of infection determined was found to be relatively robust to the precise choice of thresholds used. For further details on the algorithm and a spatio-temporal display of the result, see the electronic supplementary material.

### 2.5. Spatial models

In formulating our inter-city transmission model, we take the city as our unit of study. Each of *N* cities, *i*, has an infection time *t*_*i*_, an invariant population size *P*_*i*_ and a time-varying mortality rate, *r*_*i,t*_ at time *t*. Infected city *i* is separated from susceptible city *j* by distance *d*_*ij*_. Each week, each city can be in one of the three disease states: Susceptible, Latent or Infectious. We assume that all cities are susceptible at the start of a wave, they are latently infected for one week on infection, and that a city becomes infectious the week after it becomes infected. We assume that all transmission is endogenous to England and Wales or US after external seeding to the first infected city in each territory. If a city becomes infected in week *t*_*i*_, the candidate infectors are only those cities that are infectious in week *t*_*i*_. We assume that the transmission parameters are constant through time.

The model formulation aims to capture the effect of distance and population size on the connectivity of cities. Three modes of spatial transmission are considered: density-independent connectivity, density-dependent connectivity and an intermediate form where the degree of density dependence is estimated. The model also examines the different assumptions regarding a city's infectivity over time.

The force of infection, *λ* is the hazard of infection from one city to another. From infected city *i* on susceptible city *j* at time *t*, it is:
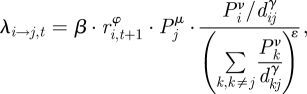
where source city population and distance are normalized together. *ν* and *μ* are estimated parameters on source and destination population sizes, respectively. *d_ij_* represents the distance between cities with power parameter *γ* to be estimated.

*φ* is an estimated parameter relating the infectivity of a city to its mortality rate. When *φ* = 1, the infectiousness of a city at time *t*_*i*_ is proportional to the death rate in that city at time *t_i_* + 1. We use one week as a lag from infection to death [24,34–36]. A value of *φ* = 0 gives a flat infectiousness profile, independent of the death rate in the source city. Intermediate values of *φ* give variation in infectiousness, which scales sub-linearly with weekly mortality. Estimating *φ* allows us to assess whether mortality rate is a good proxy for infectiousness in an infected city. *β* is a time-invariant estimated infectivity term.

Parameter *ɛ* describes the strength of connection of a susceptible city to all possible infectors. *ɛ* = 0 gives the density-dependent model and *ɛ* = 1 gives the density independent model. By allowing *ɛ* to vary, we allow the model to estimate the degree of density dependence in connectedness between the cities.

The total force of infection on city *j* at time *t* is given by:
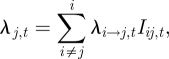


where
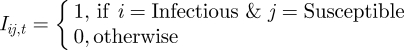


Since the force of infection is a hazard, the probability that a susceptible city *j* is infected in a week *t_j_* is given by:
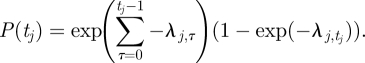


### 2.6. Estimation of parameters

We used a Bayesian framework for statistical inference. The log-likelihood is given by:
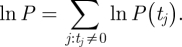


We explore the joint posterior distribution of parameters by Markov Chain Monte Carlo (MCMC) sampling [37,38]. We sampled parameters on a log-scale using a random walk update scheme. *β* and *γ* were jointly updated, while *ɛ*, *μ* and *ν* were updated singly. Five MCMC chains were started from a variety of start points within a credible range to assess convergence. Convergence was achieved within 100 000 iterations for all models from all starting parameter values. For each model, the chain was run for 500 000 iterations including a burn in of 100 000. Parameter estimates and equal-tailed 95 per cent credible intervals were obtained from the posterior distribution of 80 000 values thinned from the last 400 000 samples of the MCMC chains.

### 2.7. Model variants

To investigate which components are most important for describing the spread of influenza, we consider a set of simplified variants of the model presented above. In those variants, each parameter can be either fixed at 0, at 1 or be estimated by MCMC. For a full comparison of each component of the model, see the electronic supplementary material.

The Deviance Information Criterion (DIC) is used to compare models [39]. This is calculated using the median parameter values owing to non-normality in the likelihood [39,40]. Lower values are preferable and a difference of around 5 units is considered important [41].

### 2.8. Epidemic trees

The model is used to generate epidemic trees [42,43]. We sample 1000 parameter sets from the joint posterior distribution and calculate the probability of infection for each potential infector city. The most likely tree for each parameter set is generated by calculating which ‘infector’ city has the highest probability of infecting each ‘infectee’ city. The distance to this infector, the probability of the infector–infectee pair and the number of infectees each infector creates are calculated for each parameter set. Mean values are weighted by the frequency of infector–infectee pairs from 1000 trees.

### 2.9. Validation

We examine the ability of the models to recreate the observed epidemic by simulation. We use 1000 parameter sets sampled from the joint posterior distribution and for each set, we simulate an epidemic using the first infected city as a source of infection. Once a city is infected, the observed mortality curve is used to model the infectiousness of that city through time.

We also calculate the probability distribution of the week of infection for each city conditional upon the observed epidemic up to that time point. We use 1000 parameter sets sampled from the joint posterior distribution and for each set, we calculate the probability of infection each week for each city given the epidemic observed up to that time point. We use Welch's two-tailed *t*-test to differentiate outlying groups.

## 3. Results

### 3.1. Model components

Comparison of spatial and non-spatial population-independent models shows that inclusion of distance substantially improves model fit for both England and Wales and the US (Δ_DIC_ = 64.7 and Δ_DIC_ = 33.3, respectively). DIC and parameter estimates for the distance-only model are given in column 1 of tables 1 and 2.

**Table 1.** Parameter estimates for six models in England and Wales. Posterior medians and equal tailed 95% credible interval presented for each parameter.

	distance-only model	density-dependent population model	density-independent population model	estimated density dependence population model	full model	single-infected city parameter model
DIC	880.4	863.6	844.5	846.3	846.4	841.9
*γ* (distance power)	0.90 (0.71, 1.07)	0.85 (0.66, 1.03)	1.14 (0.92, 1.34)	1.15 (0.93, 1.36)	1.18 (0.93, 1.40)	1.18 (0.96, 1.39)
*μ* (susceptible population)	0	0.30 (0.15, 0.44)	0.36 (0.22, 0.51)	0.35 (0.19, 0.48)	0.40 (0.24, 0.53)	0.40 (0.25, 0.54)
*ν* (infected population)	0	0.20 (0.01, 0.67)	0.14 (0, 0.48)	0.15 (0, 0.50)	0.22 (0.01, 0.60)	0
*ɛ* (spatial interaction)	0	0	1	0.90 (0.64, 1.21)	0.86 (0.55, 1.24)	0.87 (0.61, 1.16)
*φ* (infectivity parameter)	0	0	0	0	0.30 (0.06, 0.62)	0.24 (0.03, 0.47)
*β* (intensity)	0.0007 (0.006, 0.008)	0.0003 (0, 0.001)	0.04 (0.01, 0.17)	0.02 (0, 0.24)	0.15 (0.01, 2.44)	0.11 (0.01, 1.01)

**Table 2.** Parameter estimates for six models in US. Posterior medians and equal tailed 95% credible interval presented for each parameter.

	distance-only model	density-dependent population model	density-independent population model	estimated density dependence population model	density-dependent population model with infectivity	distance-only with infectivity
DIC	114.5	115.3	119.1	117.2	114.8	114.8
*γ* (distance power)	0.79 (0.54, 1.01)	0.77 (0.52, 0.99)	1.04 (0.68, 1.41)	0.99 (0.65, 1.35)	0.85 (0.60, 1.10)	0.86 (0.61, 1.10)
*μ* (susceptible population)	0	0.07 (0.003, 0.28)	0.11 (0.01, 0.35)	0.10 (0.004, 0.34)	0.08 (0.004, 0.27)	0
*ν* (infected population)	0	0.75 (0.03, 1.86)	0.28 (0.01, 0.91)	0.37 (0.02, 1.32)	1.13 (0.07, 2.09)	0
*ɛ* (spatial interaction)	0	0	1	0.66 (0.15, 1.09)	0	0
*φ* (infectivity parameter)	0	0	0	0	1	1
*β* (intensity)	0.07 (0.05, 0.10)	0.03 (0, 0.11)	0.69 (0.04, 2.80)	0.22 (0.01, 1.91)	71.9 (6.26, 284)	130 (87.7, 193)

**Figure 2.**
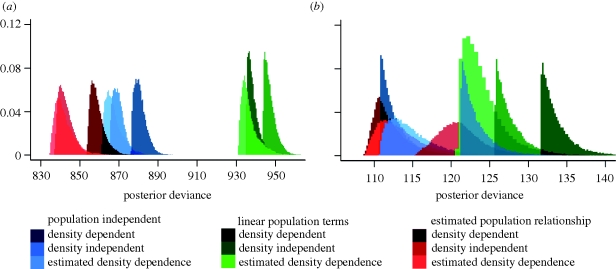
Posterior deviances of nine models for comparison in (*a*) England and Wales, and (*b*) the US. Blue models are population independent, green have a linear relationship with source and destination population size, and red estimate the relationship between the population sizes of both source and destination cities and connectivity. In each case, the darkest curve represents the density-dependent formulation, medium the density independent and the lightest is the model which estimates the degree of density dependence.

Previous formulations of the gravity kernel in the literature have considered either density-dependent (*ɛ* = 0) or density-independent transmission (*ɛ* = 1). Figure 2 compares the fit (expressed by the posterior deviance) of these two formulations and with that from the model where the degree of density dependence, *ɛ*, is estimated. This comparison is made for models assuming no linear or a fitted power-dependence of spatial coupling on both source and destination city population size. In figure 2*a*, *φ* is estimated, whereas in *b* it is fixed at 1. See model components in the electronic supplementary material for further comparisons.

For England and Wales (figure 2*a*), in each population context the variant that estimates the degree of density dependence (the lightest curve of each colour) gives a slightly better fit than models with no density dependence, with pure density dependence fitting substantially less well. The comparison also shows that the models which estimate the effect of origin and destination city population sizes on the connectivity of cities are much better than either the population-independent or linear population size-dependent models.

The same set of comparisons is made for the US in figure 2*b*. The situation is more complex with the posterior distribution of many model variants lying in the same area. Comparisons by DIC value cannot distinguish these models. Unlike in England and Wales, there is no density-dependence variant which has lower deviance for all three of the population size-dependence variants examined. Inclusion of nonlinear population size-dependence does not penalize the fit of the US model, and so cannot be definitively excluded as being consistent with the data. The models presented in columns 5 and 6 in table 2 have different population relationships, but the same DIC score. The credible intervals on the population parameters of the density-dependent population with infectivity model (column 5) are very wide suggesting that little information is added by the inclusion of these parameters.

In England and Wales, the lowest DIC model is one where the degree of density dependence is estimated and the effect of population is also estimated. This is in contrast to the US where population-independent models either with density-dependent or estimated density dependence spatial interaction terms are indistinguishable.

### 3.2. Impact of infectivity profile

We tested models with three types of infectiousness profile through time: constant infectivity, a linear relationship between infectivity and mortality in the week ahead, and an estimated power-law relationship between mortality and infectivity. Mixing was poor when estimating *φ* with the US data so we only compare the first two models in that setting.

In England and Wales, the linear infectivity model has a DIC value of more than 25 above either the constant or estimated infectivity model. Parameter estimates for the constant-infectivity and estimated-infectivity model variants are shown in columns 4 and 5 of table 1 using the density-dependent population-dependent framework from the previous comparison in England and Wales. These two models are indistinguishable by DIC (Δ_DIC_ = 0.1). Estimates for all other parameters are very comparable between these two models.

The estimated relationship includes two inputs from the infected city: the mortality rate and the population of the city. It can be more difficult to estimate parameters regarding infectivity, so we tested a model which takes only one piece of information from the infected city. The final column in table 1 shows a model which takes the mortality rate from the infected city into account but does not include the population size of that city. There is an improvement in the DIC score for this model of 4.4 over the constant infectivity model.

Table 2 shows parameter estimates for models in the US. The difference in DIC score between a constant infectivity model and one with a linear relationship between mortality and infectivity is negligible in either a distance-only model framework (columns 1 and 6) or a population-dependent framework (columns 2 and 5). Adding infectivity information does not improve the fit of the model.

**Figure 3.**
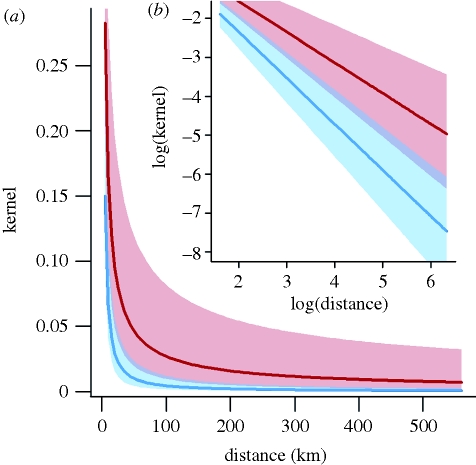
Best-fit distance kernels for England and Wales (blue) and US (red). Posterior median is shown as a darker line and 95% credible intervals are given by the shaded region. (*a*) Unlogged and (*b*) logged.

In the England and Wales dataset, the lowest DIC model is the single infected city parameter model in column 6 of table 1. The model is dependent on the destination population size, has estimated dependence of infectivity on mortality and an estimated intermediate degree of density dependence. The distance power *γ* was estimated as 1.18 (0.96, 1.39). A lower value was found for models in the US, where for the most parsimonious low DIC model (density dependent, population independent), *γ* was estimated as 0.79 (0.54, 1.00). Figure 3 shows the distance kernels for the two datasets. The credible intervals for *γ* overlap for the two datasets.

The power parameter on the destination city population, *μ*, was estimated at 0.40 (0.25, 0.54) in England and Wales. The credible intervals exclude 1, demonstrating that as population size increases, the susceptibility of the city increased more slowly.

### 3.3. Comparison between datasets

We used the posterior median parameter estimates fitted to the England and Wales dataset to calculate a likelihood value in the US dataset. By likelihood ratio test, this value was not different from the most parsimonious low DIC US model (−55.23, −57.72, *p* > 0.97). We therefore cannot reject the assumption that spread had the same characteristics in the US and England and Wales, though clearly the smaller size of the US dataset reduces inferential power.

### 3.4. Infection trees

**Figure 4.**
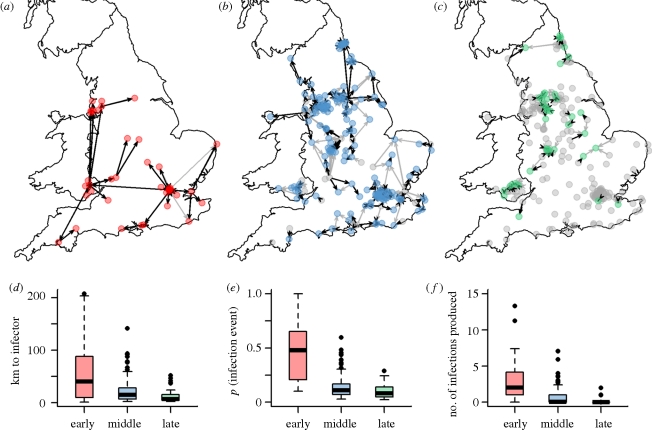
(*a–c*) The most likely infector tree for each stage of the epidemic in England and Wales. Weeks 0–4 are the (*a*) early stage of the epidemic, (*b*) weeks 5–7 are the middle and (*c*) weeks 8–10 are late in the epidemic. Black lines represent a consistent infector designation above 70% and grey shows infection events less frequent than 70% of trees. (*d–f*) comparison of the distance to inferred infector, probability of each infection event (arrows in (*a*–*c*)) and the distribution of number of new infections created by each city in each phase of the epidemic. These values are weighted for frequency within the 1000 realizations.

Figure 4 shows the most likely infection tree for each city in England and Wales stratified by the phase of the epidemic during which each city was infected. Inferred city-to-city infection events more frequent than 70 per cent (in 1000 trees) are shown in black, events of lower frequency are shown in grey. Interactions in weeks 0–3 are longer range than those in weeks 4–7 (*p* < 0.01), which are in turn longer range than those in weeks 8–10 (*p* < 0.01) (figure 4*d*). The probability that the most likely infector was responsible for each infection falls as the epidemic progresses because there are many more potential infectors available later (figure 4*e*). Cities infected early give rise to more infections than those infected late in the wave, as expected, but the range is large, with some early cities giving rise to no new infections (figure 4*f*).

**Figure 5.**
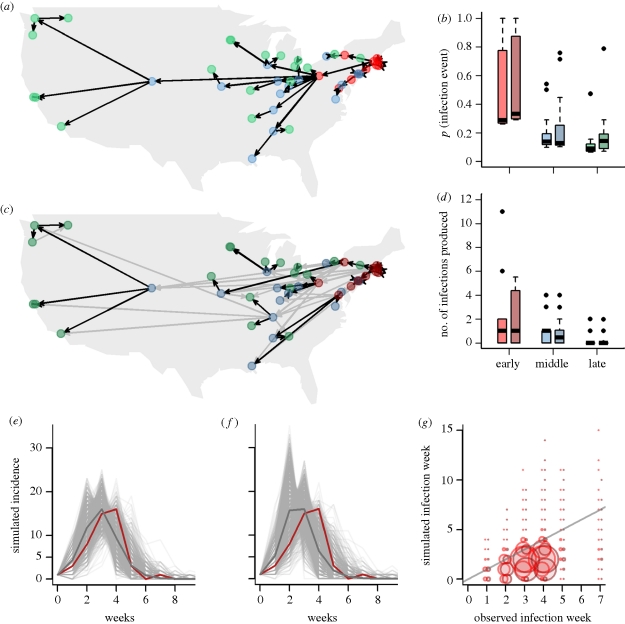
(*a*) The most likely US infection tree for 1000 parameter sets from the distance-only constant-infectivity model. Arrows show infector and infected cities. Cities infected early in the epidemic (week 0–2) are shown in red, those infected in the middle (week 3) are in blue and cities infected late in the epidemic (weeks 4–7) are shown in green. (*c*) The most likely tree for the US using 1000 parameter sets fitted to the England and Wales data. Black lines represent a consistent infector designation above 70% and grey shows any frequency of most likely infection event. (*b*,*d*) Comparison of firstly the probability of each infection event (arrows in (*a*) and (*c*)) for each stage of the epidemic, and secondly the distribution of number of new infections created by each city in each phase of the epidemic. These values are weighted by frequency within the 1000 realizations. ((*e*, *f*) The incidence curves for 1000 simulations of the epidemic from 1 start city for the distance-only constant-infectivity model (*e*) and the England and Wales parameters model (*f*)). Simulation means are shown in dark grey and the observed epidemic curve is shown in red. (*g*) The simulated week of infection against the observed week of infection for the two models. The England and Wales parameters model is darker. *r* = 0.49 and 0.48, respectively.

Figure 5 shows results for two models using the US data. We compare the most likely infection trees for the distance-only constant infectivity model with parameters inferred from the US data (figure 5*a*) with a model where parameters used to generate the trees are taken from the England and Wales single-infected city parameter model (figure 5*c*). In the distance-only constant infectivity model, the nearest infected city is always the most likely infector. In contrast, with the England and Wales parameters, some links between cities are high frequency, while other cities have several potential infectors of intermediate frequency (figure 5*b*). As in England and Wales, infection events inferred early in the epidemic have a higher support than those later in the epidemic. There are some exceptions owing to the distribution of cities in the US dataset—Oakland and San Francisco are distant from all other cities but very close to each other. In the distance-only constant infectivity model, some cities may give rise to a large number of new infections (e.g. Pittsburgh gives rise to nearly a quarter of infections) (figure 5*d*). The effect of a city acting as a hub of infection is reduced in the more complex model, as the risk of infection from one city to another is the combined effect of several factors including distance.

### 3.5. Validation

**Figure 6.**
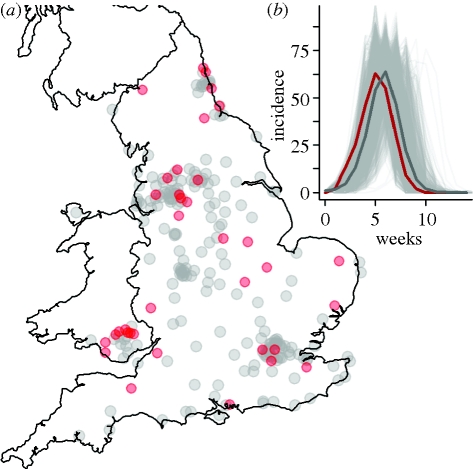
(*a*) Cities that lie outside of the 75% probability interval for infection week are shown in red. (*b*) 1000 simulations from the best model—single infected-city parameter model in England and Wales. Dark grey is the simulation mean, red is the observed epidemic curve.

For England and Wales, there is a relatively good agreement between observed and simulated epidemic curves (figure 6*b*). The observed epidemic curve rises more steeply than the simulation curves in the early stages of the epidemic, and peaks one week earlier than the simulation mean. This suggests that the model may underestimate the external infection pressure early in the wave.

We calculated the probability that a city was infected in each week given the observed behaviour of all other cities up to that time. In England and Wales, 245 of 246 cities lie within the 95 per cent interval of their expected distribution. Figure 6*a* shows the cities which the observed infection week lies outside the stricter inter quartile interval. For further information see the electronic supplementary material. There are no population size (*p* = 0.36) or density trends (*p* = 0.11) in these cities, which are typically infected later in the epidemic (*p* < 0.01 for difference in infection week). In the US, all cities lie within the 95 per cent probability interval and all but three lie within the inter quartile interval. Those three outlier cities are smaller than other cities (*p* = 0.01) but equally distributed in space (*p* = 0.88) and time (*p* = 0.06).

We have tested the effect on parameter estimates in England and Wales of relaxing the single-introduction assumption inherent in the model. We re-estimated the parameters conditioning on infections that occurred from week 3 of the epidemic onward. There is a small increase in the kernel power parameter estimate, which causes the kernel to decay more rapidly with distance (electronic supplementary material, figure S9). This suggests that the very long-range interactions, which are forced to occur early in the epidemic impact the shape of the kernel. However, the credible intervals largely overlap which indicates this assumption does not affect the fit of the model to a large degree.

In the US the simulated curves for the distance-only constant infectivity model are shown in figure 5*e* and for the England and Wales parameters in figure 5*f*. In both cases, the mean simulated and observed curve are very comparable, with the distance-only constant infectivity model giving peak incidence in the same week as observed. Figure 5*g* shows the observed week of infection against the simulated week of infection for all 1000 simulated epidemics. There is good correlation between the observed and simulated weeks of infection for both parametrizations.

We have tested the effect of thinning the England and Wales dataset so that it more closely resembles the US dataset to determine if the differences in formulation between the best models for each dataset are owing to the smaller number of cities in the US dataset. We removed all cities with fewer than 90 000 inhabitants in England and Wales leaving 46 cities distributed quite evenly in England and Wales as shown in the electronic supplementary material, figure S10. There were identifiability problems in estimating the density-dependence parameter *ɛ* using the thinned dataset. The best model by DIC comparison gave a distance-only interaction (no dependence on population size) with infectivity scaling linearly with mortality in a density-independent framework. As we found with the US data, it is difficult to disentangle the effects of population and infectivity parameters because these feature in different combinations in comparable DIC models.

## 4. Discussion

We have presented a statistical analysis of the spatio-temporal spread of the 1918 influenza pandemic between cities in England and Wales and the US. The results demonstrate that for England and Wales, a model with intermediate levels of density dependence in the connectivity between cities gives the best fit to the observed pattern. For the US dataset, where there are few, large and widely spaced population centres, estimating the degree of density dependence does not improve the fit. In both contexts, city population size affects inter-city coupling sub-linearly. Parameter estimates and model formulation inferred from the data of England and Wales explain the US dataset well. Gravity model parameter estimates generated in this study are comparable with values found in studies describing the spread of seasonal influenza [5,44].

Our analysis demonstrates the degree of spatial locality in the large-scale geographical spread of influenza in both England and Wales and the US in 1918. However, it is difficult to directly compare the kernel power estimate from this study with those from other studies owing to differences in the functional forms used. For instance, Viboud *et al.* [5] estimate two power parameters above and below a given distance threshold when modelling the spread of seasonal influenza in the US. Gravity models used to describe the spread of measles in the UK by Xia *et al.* [18] assumed a kernel power of 1, rather than fitting this parameter. The distance power estimates we found for England and Wales and the US are quite different from each other. It is not surprising that there is a disparity in the distance kernel in England and Wales and the US, as the spatial scale in the US is much larger than in England and Wales. In comparing the US and UK, it should be noted that the mean distance between cities is of course much larger in the US (see electronic supplementary material, table S1). In theory, this gives better resolution for estimating the kernel shape, as the range of inter-city separations is an order of magnitude larger than for the UK. However, this is counterbalanced by the smaller size of the US dataset, which reduces inferential power. The low kernel power parameter estimates we have found in both England and Wales and the US suggests that long-distance interactions were important in spreading influenza between distant cities in both countries. At the start of the major autumn wave in 1918, the armistice was more than two months away and it is likely that travel relating to the war effort, including troop movements, might have enhanced the frequency of long-distance movements.

Density-dependent gravity models are frequently used to explain the connectivity of urban centres for human diseases [5,18]. There is good evidence from the estimates of *ɛ* in this analysis for England and Wales that the density-dependent model underestimates the total force of infection on remote cities. The 95 per cent credible interval for *ɛ* includes 1, which indicates that the density-independent model formulation cannot be definitively excluded as an explanation for the data. There was limited statistical power to estimate the degree of density dependence in the US context, but use of the England and Wales best-fit model to describe the US data gave a very similar DIC to the best-fit US model. Hence, it is not clear if the difference in the estimated density dependence found between the US and the England and Wales is because of the large differences in the degree of population coverage between the US and England and Wales datasets. Our results using a subset of the England and Wales dataset suggest that the degree of density dependence is a difficult parameter to estimate when coverage is low.

The low power on destination city size found in fitting the gravity model to the England and Wales dataset shows that connectivity of a city increases sub-linearly with population increase. When modelling spread of influenza in the US, Viboud *et al.* [5] found very comparable low values of the population exponents with the infectious city lower than the susceptible. Differing results come from the analysis of measles data in Great Britain with a power coefficient on infectious populations estimated at approximately 1.5 [18]. We found the best-fit model in England and Wales does not include the population size of the infector (origin) city, a result which needs further examination in future work. Differences in our population parameter estimates and those from studies on contemporary populations are likely to differ owing to changes in human mobility patterns since 1918.

Our estimate for England and Wales that the infectivity of a city is sub-linearly related to mortality, suggests that the rate of death in a city is not as important to infectivity as the presence or absence of disease. Other studies have used constant infectivity terms for the analysis of human seasonal influenza [21]. However, our estimates do support some level of mortality dependence, suggesting that cities with a very high influenza burden, usually later in the epidemic wave, are more infectious than newly infected cities. In the US dataset, the best-fit model gave constant infectiousness, but again this may be due to a lack of power to estimate such parameters from the US dataset. It may also be caused by non-uniform infection pressure from cities not in the dataset, which could mask an infectivity relationship for the cities that are given.

Future possible extensions of this work include relaxing the assumption that all cities were equally susceptible at the start of the autumn wave of the pandemic. The variation in the onset of infection in cities may, in part, be due to the differing susceptibility of each city owing to differing attack rates experienced in the spring–summer wave, or population-level immunity from the 1890 pandemic or seasonal strains. However, the low case fatality of the first wave and age-specificity of infection between waves need to be understood before spatial heterogeneity in susceptibility can be discerned. There are varying reports on the magnitude and mechanism of the effect of infection during the first wave on attack rates in subsequent waves [24–26,45–47]. Further analysis of the datasets considered here may provide an opportunity to disentangle these effects.
